# Tamoxifen Suppresses the Immune Response to *Plasmodium berghei* ANKA and Exacerbates Symptomatology

**DOI:** 10.3390/pathogens10060743

**Published:** 2021-06-12

**Authors:** Luis Antonio Cervantes-Candelas, Jesús Aguilar-Castro, Fidel Orlando Buendía-González, Omar Fernández-Rivera, Armando Cervantes-Sandoval, Jorge Morales-Montor, Martha Legorreta-Herrera

**Affiliations:** 1 Unidad de Investigación Química Computacional, Síntesis y Farmacología de Moléculas de Interés Biológico, Laboratorio de Inmunología Molecular, Facultad de Estudios Superiores Zaragoza, Universidad Nacional Autónoma de México, Ciudad de México 09230, Mexico; cervantescandelasluis@gmail.com (L.A.C.-C.); jesus_aguilar_castro@yahoo.com.mx (J.A.-C.); fidel_zzz@hotmail.com (F.O.B.-G.); qfbfdz@gmail.com (O.F.-R.); 2 Posgrado en Ciencias Biológicas, Unidad de Posgrado, Edificio D, 1° Piso, Circuito de Posgrados, Ciudad Universitaria, Coyoacán, Ciudad de México 04510, Mexico; 3 Laboratorio de Aplicaciones Computacionales, Facultad de Estudios Superiores Zaragoza, Universidad Nacional Autónoma de México, Ciudad de México 09230, Mexico; arpacer@unam.mx; 4 Departamento de Inmunología, Instituto de Investigaciones Biomédicas, AP 70228, Universidad Nacional Autónoma de México, Ciudad de México 04510, Mexico; jmontor66@biomedicas.unam.mx

**Keywords:** malaria, *Plasmodium berghei* ANKA, tamoxifen, oestrogens, immune system

## Abstract

Malaria is the most lethal parasitic disease in the world. Mortality and severity in symptoms are higher in men than women, suggesting that oestrogens, which are in higher concentration in females than in males, may regulate the immune response against malaria. Tamoxifen, a selective oestrogen receptor modulator used in breast cancer treatment due to its antagonistic effect on oestrogen receptors α and β, is also studied because of its potential therapeutic use for several parasitic diseases. However, most studies, including one in malaria, have not addressed the immunomodulatory role of tamoxifen. In this work, we evaluated the effect of tamoxifen on the immune response of CBA/Ca mice against *Plasmodium berghei* ANKA. This study showed for the first time that tamoxifen increased parasite load, aggravated symptoms by decreasing body temperature and body weight, and worsened anaemia. Additionally, tamoxifen significantly increased the splenic index and the percentages of CD4^+^ and NK^+^ cells on day eight post-infection. By contrast, tamoxifen decreased both CD8^+^ and B220^+^ populations in the spleen and decreased the serum levels of IL-2, IL-6, and IL-17. Our findings support the notion that tamoxifen is a potent immunomodulator in malaria-infected mice and suggest caution when administering it to malaria-infected women with breast cancer.

## 1. Introduction

As the most lethal parasitic disease globally, malaria was responsible for 409,000 deaths in 2020 alone and 229 million new cases [1]. In epidemiological studies and experimental malaria models, males exhibit higher mortality, parasitaemia, increased anaemia, and stronger weight loss than females [2,3]. These findings indicate that oestrogens contribute to this sexual dimorphism [4]. These molecules constitute the major sex steroids in females and work as immunomodulators [5,6]. The interaction of oestrogens with their receptors activates signalling pathways such as NF-κB, ERK/MAPK, PI3K/AKT, etc., leading to activation or inhibition of transcription factors that modulate the expression of different genes [7,8,9], including those for the immune response. Oestrogens physiological levels induce the proliferation of CD4^+^ and CD8^+^ T cells which promote the synthesis of IFN-γ, TNF-α and IL-6 [10,11]. In addition, oestrogens induce the proliferation of B lymphocytes and their maturation to plasma cells, which increases the synthesis of antibodies [5,12]. Oestrogens also enhance the proliferation of dendritic cells and macrophages, which promote phagocytosis and secretion of IL-6, TNF-α and IFN-γ [13,14]. In addition, it has been shown that oestrogens modulate the immune response in several parasitic diseases [15,16,17].

We have previously shown that gonadectomy (which eliminates the primary source of oestrogens, the ovaries) detrimentally affects the immune response in females infected with *P. berghei* ANKA; it increases TNF-α and IL-6 serum levels but decreases the mRNA expression of IFN-γ in blood [18]. In addition, 17β-oestradiol administration to intact female mice infected with *P. berghei* ANKA increases parasitaemia and decreases body weight. By contrast, reconstitution of gonadectomised female mice using 17β-oestradiol reduces parasitaemia and affects the immune response [19,20].

On the other hand, tamoxifen, a selective oestrogen receptor modulator, used in the treatment of breast cancer due to its antagonistic effects on α- and β-oestrogen receptors [21]; it also has immunomodulatory effects [22]. Interestingly, several studies have focused on its possible therapeutic use in several parasitic infections. The tamoxifen administration to mice infected with *Taenia crassiceps* reduced parasite load, decreased its reproduction and loss of motility in female mice [23]. In vitro, tamoxifen inhibits the evagination of *Taenia solium* cisticerci, and in vivo, it decreased the intestinal establishment of this parasite in hamsters [24]. Additionally, tamoxifen also inhibits the survival of *Echinococcus granulosus* in vitro and protect mice against its infection [25]. Furthermore, tamoxifen induces morphological alterations in *Schistosoma mansoni* parasites and eggs in vitro [26].

Tamoxifen also exhibited anti-protozoan activity in vitro against *Trypanosoma cruzi*; induced lysis and triggered an apoptotic death process of trypomastigotes in vitro [27]. Moreover, tamoxifen killed *Leishmania amazonensis*, *Leishmania branziliensis*, *Leishmania major*, *Leishmanida donovani* and *Leishmania chagasi* in vitro [28]. Tamoxifen topical therapy was also efficient in decreasing lesion size and parasite load in an experimental model of cutaneous leishmaniasis [29]. Finally, tamoxifen has also been used in malaria, but its anti-parasitic activity has led to controversial results. Tamoxifen has been reported to have antimalarial activity in vitro and in vivo against *Plasmodium falciparum* and *Plasmodium berghei*, respectively [30]. However, no antimalarial activity was detected in Swiss mice infected with a *Plasmodium yoelii nigeriensis* isolate (*Py-R2*) [31].

Given that oestrogens modulate the immune response, particularly in malaria, that tamoxifen, an oestrogen receptor antagonist, also exhibits an immunomodulatory and parasiticidal activity for various nematodes and protozoa. In this work, we decided to study the effect of tamoxifen on *Plasmodium berghei* ANKA proliferation in vivo and its effects on the immune response in CBA/Ca mice. We assessed parasite load, splenic index, number of immune response cells in the spleen and circulating levels of pro- and anti-inflammatory cytokines.

## 2. Results

### 2.1. Tamoxifen Increased Parasitaemia and Splenic Index of CBA/Ca Mice Infected with Plasmodium berghei ANKA

To assess the effects of tamoxifen on parasitaemia and inflammation in the spleen. CBA/Ca mice were treated with tamoxifen and then infected with *Plasmodium berghei* ANKA. We used controls groups of infected mice treated only with the vehicle or infected without treatment. To demonstrate that the effects are due to tamoxifen and not to infection with the parasite, we used three additional groups of mice treated in the same way but without infection sacrificed on the day corresponding to day eight post-infection.

**Figure 1:**
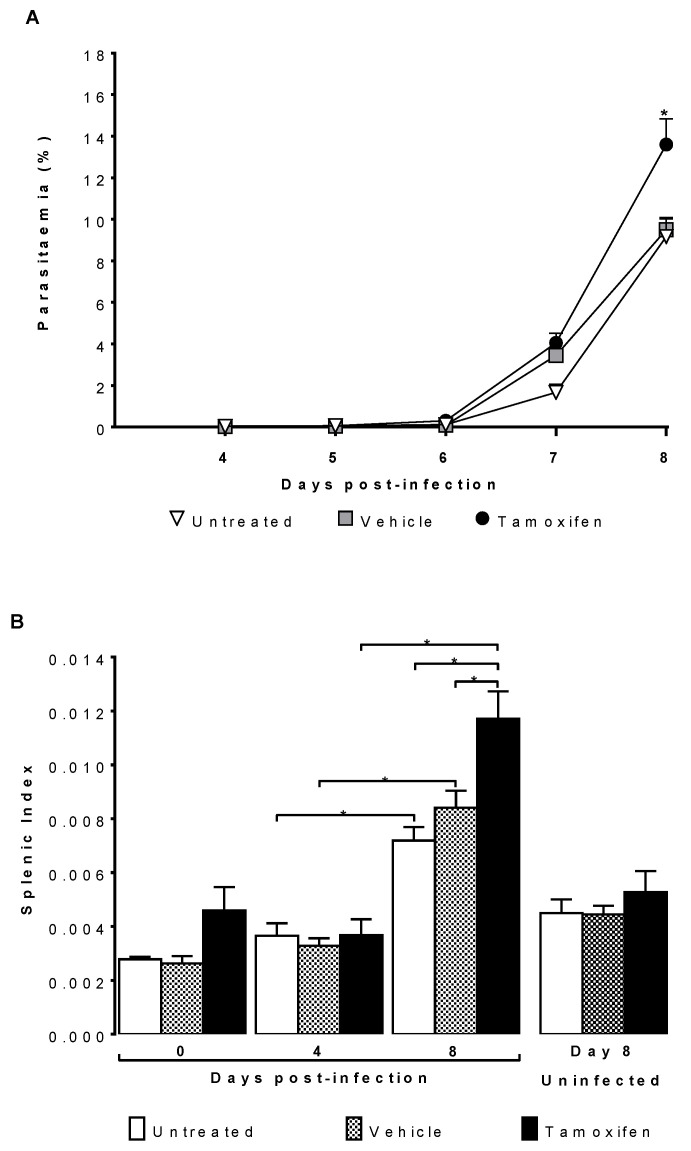
Tamoxifen increased parasitaemia and spleen index of CBA/Ca mice infected with *Plasmodium berghei* ANKA. Groups of CBA/Ca female mice were treated with either tamoxifen or vehicle or left untreated as controls; all groups were infected with *Plasmodium berghei* ANKA. (**A**) The percentage of parasitised erythrocytes was evaluated daily; each point represents the geometric mean of parasitaemia in each group (*n* = 10). (**B**) Mice were sacrificed at 0, 4 and 8 days post-infection; the graph shows the spleen index; each bar represents the mean ± SEM in each group (*n* = 10). Additionally, three control groups treated in the same way as the mice on day eight but without infection (*n* = 10) were analysed. Asterisks represent the statistically significant difference between the groups (*p* ≤ 0.05) using repeated means test for parasitaemia and nested analysis test for splenic index, both with post hoc Bonferroni test.

Tamoxifen significantly increased parasitaemia on day eight post-infection compared to that in both control groups, the untreated and the vehicle-treated groups (Figure 1A). The splenic index increased as the infection progressed, reaching its higher level on day eight post-infection in all groups. Of note, the groups infected treated with tamoxifen exhibited the highest splenic index compared to the groups treated with vehicle or untreated. As expected, the uninfected mice did not significantly increase the splenic index; however, the tamoxifen-treated groups tended towards a higher splenic index (Figure 1B).

### 2.2. Tamoxifen Aggravates Pathology by Decreasing Body Temperature, Body Mass, and Haemoglobin Levels in CBA/Ca Mice Infected with Plasmodium berghei ANKA

**Figure 2:**
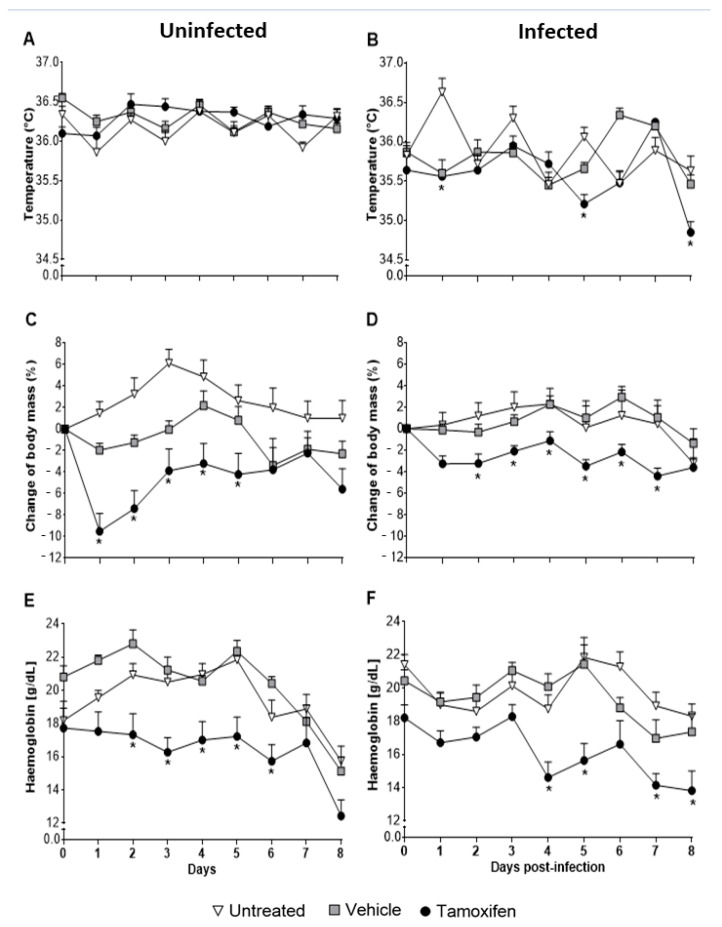
Tamoxifen decreased the temperature, body mass, and haemoglobin concentration of CBA/Ca mice infected with *Plasmodium berghei* ANKA. Groups of mice treated with tamoxifen, vehicle or untreated, were used to evaluate its effect on body temperature, body weight and haemoglobin concentration. All variables were assessed daily. Panels A, C and E correspond to the uninfected control groups, while graphs (B, D and F) correspond to mice infected with *P. berghei* ANKA. (**A**) represents body temperature in uninfected mice, while (**B**) represents temperature in mice infected with *P. berghei* ANKA. (**C**,**D**) represent the change in body weight in uninfected and infected mice, respectively. Finally, (**E**,**F**) represent the haemoglobin concentration in uninfected and *P. berghei* ANKA infected mice, respectively. Each line represents the mean ± SEM in each group for the indicated days. Asterisks represent statistically significant differences between the tamoxifen-treated and vehicle-treated groups, *p* ≤ 0.05, (*n* = 10); data were analysed using repeated means tests with a Bonferroni post hoc test.

Administration of tamoxifen to uninfected mice significantly decreased body mass and haemoglobin concentration but did not change temperature (Figure 2A,C,E). While in the infected mice, the body temperature decreased significantly compared to the infected group treated with vehicle and the infected group without treatment (*p* ≤ 0.05) (Figure 2B). In addition, infected mice treated with tamoxifen significantly decreased body mass during all the experiment (Figure 2D) and significantly decreased haemoglobin concentration compared with the untreated group (*p* ≤ 0.05) (Figure 2F).

### 2.3. Tamoxifen Decreased the Percentage of Immune Response Cells in the Spleen of Mice Infected with P. berghei ANKA

**Figure 3:**
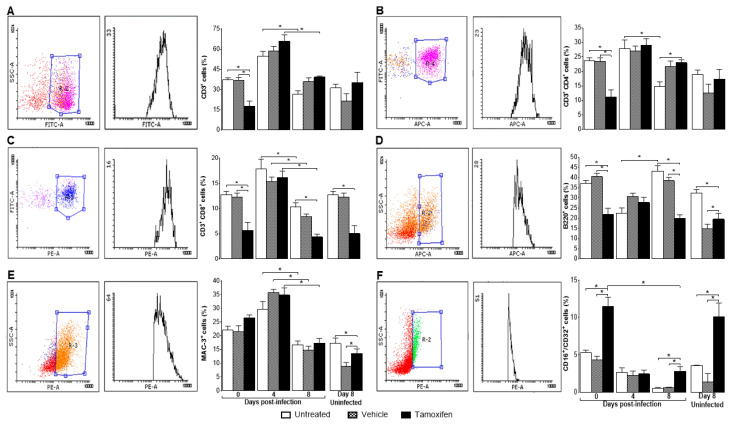
Tamoxifen decreased CD8^+^ and B220^+^ and increased NK cells in the spleen of *P. berghei* ANKA infected mice. Groups of female mice were treated with tamoxifen or vehicle or untreated as controls, were infected with *Plasmodium berghei* ANKA. At 0, 4, and 8 days post-infection, mice were sacrificed, and their spleens cells were stained with specific monoclonal antibodies and analysed by flow cytometry. Additionally, three control groups treated in the same way as the mice on day eight but uninfected (*n* = 10) were analysed as controls of tamoxifen effects in the absence of infection. The first box provides an example of how each cell population was selected according to the fluorescence emitted by the fluorochrome coupled to the specific antibody for that population and the cell complexity. The second box represents the histogram for each population, and the bars represent the mean ± SEM of the percentage of cells in each group. (**A**) CD3^+^ lymphocytes; (**B**) CD3^+^CD4^+^ lymphocytes; (**C**) CD3^+^CD8^+^ lymphocytes; (**D**) B220^+^ (B lymphocytes); (**E**) Mac3^+^ (macrophages); (**F**) CD16^+^/CD32^+^ (NK cells). Asterisks represent statistically significant differences between groups, (*p* ≤ 0.05), (*n* = 10) using a nested analysis test with a Bonferroni post hoc test.

Tamoxifen showed immunomodulatory activity even in uninfected mice; on the day of treatment that would correspond to day eight post-infection, tamoxifen significantly decreased the percentage of CD8^+^ cells and significantly increased the CD16^+^/CD32^+^ cells (Figure 3C,F), respectively.

On day zero post-infection, tamoxifen significantly decreased CD3^+^, CD3^+^/CD4^+^, CD3^+^/CD8^+^ and B220^+^ cells compared with both control groups (Figure 3A–D). However, tamoxifen did not affect the percentage of macrophages (MAC3^+^) (Figure 3E). In contrast, tamoxifen significantly increased NK^+^ (CD16^+^/CD32^+^) cells (Figure 3F). In general, infection increased all cell populations on day four post-infection except for the B220^+^ and CD16^+^/CD32^+^ cells; both populations decreased (Figure 3D,F). While on day eight post-infection, CD3^+^, CD3^+^/CD4^+^, CD3^+^/CD8^+^ and MAC-3^+^ cells decreased significantly (*p* ≤ 0.05) compared with their respective counterpart group on day four post-infection (Figure 3A–C,E). However, on day eight post-infection, tamoxifen significantly decreased the percentage of B220^+^ (Figure 3D), it did not modify the percentage of macrophages (Figure 3E), but increased NK^+^ cells compared with both control groups (*p* ≤ 0.05) (Figure 3F).

### 2.4. Tamoxifen Modified Plasma Levels of Th1, Th2 and Th17 Cytokines from CBA/Ca Mice Infected with Plasmodium berghei ANKA

**Figure 4:**
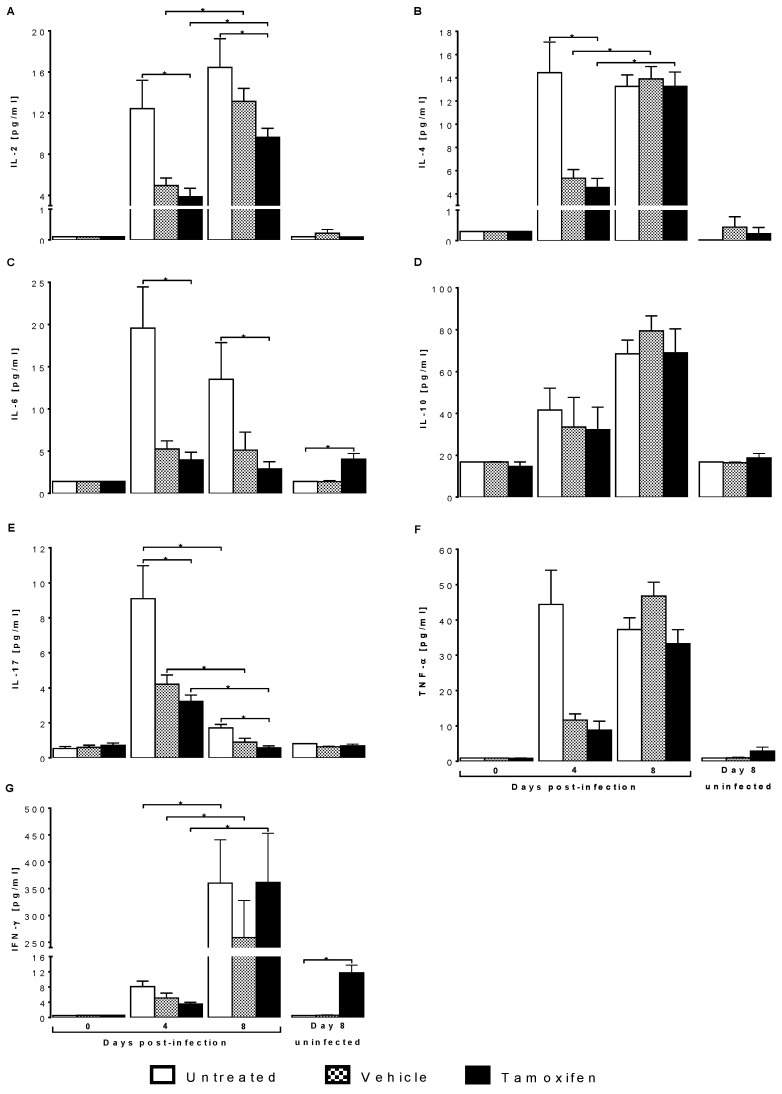
Tamoxifen decreased the levels of IL-2, IL-6, and IL-17 in CBA/Ca mice infected with *Plasmodium berghei* ANKA. Groups of female CBA/Ca mice were treated with tamoxifen or vehicle or untreated as controls, then were infected with *Plasmodium berghei* ANKA. Additionally, three control groups treated in the same way as the mice on day eight but uninfected (*n* = 10) were analysed as controls of tamoxifen effects in the absence of infection. The histograms represent the plasma concentration of the following cytokines: (**A**) IL-2, (**B**) IL-4, (**C**) IL-6, (**D**) IL-10, (**E**) IL-17, (**F**) TNF-α and (**G**) IFN-γ 0, 4, and 8 days post-infection. The bars represent the means ± SEM in each group. Asterisks represent the statistically significant difference among the groups, *p* < 0.05, using a nested analysis test (*n* = 10) and a post hoc Bonferroni test.

Tamoxifen significantly increased IFN-γ concentration in uninfected mice. On day zero post-infection, all cytokines exhibited a shallow concentration; no differences were detected between the groups. On day four post-infection, the concentration of all cytokines increased significantly (*p* ≤ 0.05) compared to day zero post-infection, particularly in the infected untreated group (Figure 4A–G). On day eight post-infection, tamoxifen increased IL-2, IL-4, TNF-α and IFN-γ compared to the same group on day four (Figure 4A,B,F,G). Interestingly, tamoxifen increased IL-6 and IFN-γ in uninfected mice (Figure 4C,G).

## 3. Discussion

For the first time, we show that tamoxifen, far from being parasiticidal, increases parasite load in *P. berghei* ANKA-infected mice. This drug also worsens the symptomatology by increasing the splenic index and decreasing haemoglobin concentration, body temperature and body weight. In addition, tamoxifen differentially affected immune response cells; it decreased CD3^+^CD8^+^ and B220^+^ cell populations and increased the number of NK^+^ cells. Finally, tamoxifen decreased circulating concentrations of IL-2, IL-6 and IL-17.

The interaction of oestrogens with their receptors activates different signalling pathways that modulate the expression of immune response genes [32]. We recently showed that parasitaemia of *Plasmodium berghei* ANKA-infected mice varies depending on 17β- oestradiol concentration [20]. In this work, we found that tamoxifen, an estrogen receptor binding antagonist, increased parasitaemia. In contrast, Weinstock et al. showed that tamoxifen and its primary metabolite 4-HO-tamoxifen decreases parasitaemia in mice infected with *Plasmodium berghei* ANKA [30]. A possible explanation for this difference is the time and dose of tamoxifen used, as Weinstock et al. administered a dose four times higher than ours. In addition, the strain of mice also generated differences in parasitaemia levels [33]. Weinstock group used a C57Bl/6 mutant strain of mice, and we used CBA/Ca mice.

That tamoxifen showed no antimalarial activity in CBA/Ca mice is in sharp contrast to other parasitic infections such as taeniasis [23], schistosomiasis [26], leishmaniasis [34] and trypanosomiasis [27]. The possible reasons for this result are diverse and complex; one possible explanation is that some parasites produce oestrogenic molecules [35], and tamoxifen interferes with the metabolism of both parasites and host [36]. Additionally, in in vivo studies, the parasite may be located inside different cells or organs, making tamoxifen inaccessible or requiring higher doses to be effective. It is also likely that both the parasite and the host metabolise the drug or dilute its effect by binding to other proteins or other possible receptors [37]. This could explain why we did not detect antimalarial activity and underlines the importance of validating the therapeutic effects of antimalarials in vivo.

In addition, it has been demonstrated that *P. berghei* infection causes splenomegaly by massive recruitment of mesenchymal stem cells to the spleen [38]. In this work, tamoxifen further increased the splenic index, particularly on day eight post-infection. This finding could be explained by the fact that by increasing parasitaemia, tamoxifen could increase antigenic stimulation in the spleen, promoting inflammation and cell recruitment in the spleen, as described by Del Portillo et al. and Zhao, et al. [39,40].

The increment in parasitaemia leads to hypothermia, weight loss, and anaemia in rodent malaria models [41,42,43]. All these disease complications were worsened in mice treated with tamoxifen. In fact, this drug caused weight loss and decreased haemoglobin concentration even in uninfected mice. One possible explanation is that tamoxifen decreases appetite and increases fat mobilisation in rats and mice [44,45,46]. In addition, it is likely that increased parasitaemia also contributed to higher erythrocyte destruction, which decreased haemoglobin concentration. Another possible explanation is that the metabolism of tamoxifen generated 4-OH-tamoxifen, which by binding to erythrocytes, increased its permeability and promoted lysis, as described by Cruz Silva in humans [47]. Our results show tamoxifen aggravates symptomatology in *P. berghei* ANKA-infected mice.

**Figure 5:**
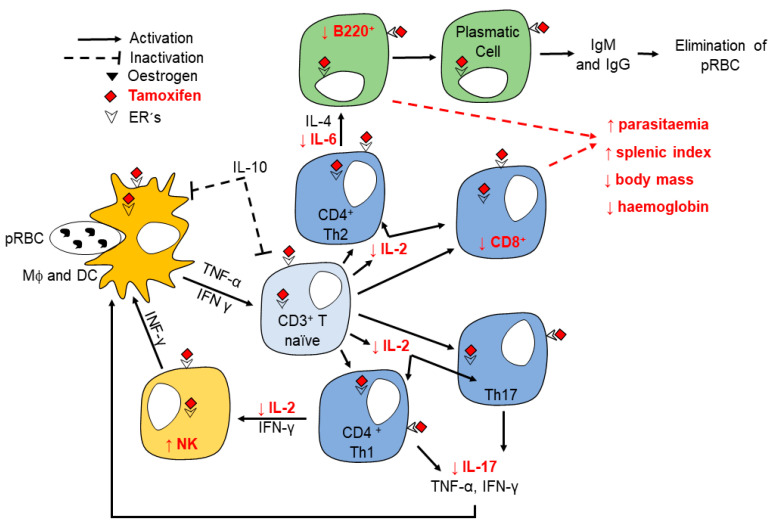
Effect of tamoxifen on the immune response of CBA/Ca mice infected with *Plasmodium berghei* ANKA. Innate and adaptive immune responses are required to control erythrocyte infection with *Plasmodium*. Dendritic cells (DC) and macrophages (Mϕ) phagocytose parasitised erythrocytes, then present parasite antigens to virgin CD3^+^; such interaction activates them to produce IL-2, promoting their proliferation and maturation to CD4^+^ Th1 cells. These last cells produce IFN-γ, which activates Mϕ and NK cells, promoting parasite elimination. On the other hand, CD4^+^ cells produce IL-4 and IL-6, which are growth and differentiation factors for B220^+^ cells to mature into antibody-producing cells involved in parasite elimination. Tamoxifen effects are labelled in red. This drug increased the number of NK^+^ cells but decreased the CD3^+^CD8^+^ population; it also decreased the concentration of IL-2, IL-6, and IL-17, which could affect parasite clearance leading to increasing parasitaemia and the splenic index as well as decreasing body mass and haemoglobin concentration.

Since the spleen is the main organ where *Plasmodium* is eliminated [35], we analysed the effect of tamoxifen on the immune response cells in the spleen. Tamoxifen decreased the populations of CD3^+^/CD8^+^ cytotoxic T lymphocytes and B220^+^ lymphocytes but increased the CD16^+^/CD32^+^ (NK) cells on day eight post-infection. The effects of tamoxifen on the immune response to malaria are indicated in red (Figure 5). In addition, tamoxifen decreased the concentration of IL-2, which is a cell differentiation and growth factor for T lymphocytes [48], which could explain the decrease in this cell population. Moreover, tamoxifen also reduced IL-6, which is a cytokine required to activate B cells as described in C57BL/6J mice infected with *Plasmodium* [49]. Therefore, possibly the decreased IL-6 concentration induced by tamoxifen would reduce the number of B cells.

Additionally, tamoxifen also decreased IL-17 levels; interestingly, low IL-17 concentration is associated with severe malarial anaemia and multiple organ dysfunction [50,51,52]. Taken together, the alterations produced by tamoxifen in immune response cells as well as cytokines, at least, partly explain the worsening of malaria symptoms when tamoxifen is administered to *P. berghei* ANKA infected mice.

Although tamoxifen exhibits activity against different parasites, attention should be observed to its therapeutical use since it is not specific and may have effects other than those expected. In the case of malaria, it promoted the proliferation of *Plasmodium* and exacerbated symptomatology. Since tamoxifen is still used in breast cancer therapy, caution is suggested when administering tamoxifen to patients with breast cancer and malaria.

## 4. Materials and methods

### 4.1. Mice and Parasites

The mouse strain used (CBA/Ca) was initially donated by Dr William Jarra (National Institute for Medical Research, London, UK). The mice were reproduced, fed, and kept under a specific pathogen-free environment at the FES Zaragoza, UNAM animal house facilities. For the experiments, 12-week-old female mice were used. The animals were kept in the animal house at the FES Zaragoza-UNAM, in strict agreement with the institutional and national official guideline NOM-062-ZOO-1999, certificate number 28/04/SO/3.4.1 for the use and care of laboratory animals.

*Plasmodium berghei* ANKA was also generously donated by Dr William Jarra. Parasites were cryopreserved under liquid nitrogen. For parasite activation, a vial was thawed and immediately injected into one mouse. When parasitaemia reached 20%, parasitised blood was extracted. Blood was diluted to infect the experimental groups of mice using an intravenous inoculation of 1 × 10^3^ *Plasmodium berghei* ANKA-infected red blood cells in each mouse.

### 4.2. Experimental Design

Ninety CBA/Ca mice were divided into three groups; the first group was untreated, the second group was administered vehicle (1% alcohol diluted in saline), and the third group was administered tamoxifen diluted in the vehicle. All groups were infected with *Plasmodium berghei* ANKA. In addition, three groups of uninfected mice treated in the same way as the mice on day 8 (*n* = 10) were used as controls of tamoxifen effects in the absence of infection.

Body temperature, body weight and haemoglobin concentration were assessed daily. Ten mice from each group were sacrificed at 0, 4, and 8 days post-infection. On the days of sacrifice, blood and spleen were removed. Plasma was separated from the blood by centrifugation and stored in aliquots to quantify the concentration of Th1, Th2 and Th17 cytokines by flow cytometry. The spleen of each mouse was weighed and disaggregated, and cells were stained with fluorochrome-labelled monoclonal antibodies and analysed by flow cytometry.

### 4.3. Tamoxifen Administration

Mice were treated daily with tamoxifen (Sigma Aldrich, St Louis, MO, USA) at a dose of 1 mg/kg body weight. We had previously calibrated the dose to achieve in vivo parasiticidal activity against *Taenia* [24]. Tamoxifen was diluted in a mixture of 1% ethanol in isotonic saline (vehicle). It was administered daily subcutaneously for 28 days before infection and for 8 days post-infection to achieve constant concentrations throughout the experiment.

### 4.4. Parasitaemia

A drop of tail blood was used daily to prepare thin blood smears, fixed with absolute methanol, and stained with 1:10 dilution of Giemsa stain (Sigma Aldrich, St Louis, MO, USA). Parasite load was assessed by light microscopy using a 100× magnification in oil immersion lens using a Carl Zeiss Standard 20 microscope (Carl Zeiss LTD, Welwyn Garden City, UK). Parasitaemia was assessed by counting the number of parasitised red blood cells compared to the total red blood cells tested (200).

### 4.5. Body Temperature

The body temperature was recorded daily at the same time using an infrared thermometer (Thermofocus, 01500A/H1N1, Vedano Olana-Varese, Italy). Each mouse was immobilised, and the infrared beam was directed 5 cm from its abdomen.

### 4.6. Body Mass Variation

Each mouse was weighed daily using an electronic balance (Ohaus, Parsippany, NJ, USA); the weight recorded on the day of infection (day zero) was considered 0%. The percentage of weight variation (PWV) was calculated daily by the following equation: PWV = ((A/B) × 100) − 100, where A is the weight of each mouse on a particular day, and B is the weight on day zero post-infection.

### 4.7. Haemoglobin (Hb) Levels

Daily, 2 µL of tail blood were mixed with 498 µL of Drabkin reagent (Sigma-Aldrich, St Louis, MO, USA) and incubated for 5 min at room temperature in the dark. The absorbance was measured at 540 nm in a microplate reader (Multiskan GO, Thermo Fisher Scientific, Inc, Walthman, MA, USA). The Hb concentration was calculated using a commercial Hb standard (Sigma-Aldrich, St Louis, MO, USA).

### 4.8. Splenic Index

At 0, 4 and 8 days post-infection, mice were weighed on an electronic balance (Ohaus), sacrificed, and the spleen was removed and weighed on an analytical balance (Sartorius, Göttingen, Germany). The splenic index was calculated as the ratio of the spleen weight and body mass for each mouse.

### 4.9. Quantification of Spleen Cell Populations by Flow Cytometry

The quantification of cell populations was assessed by multicolour flow cytometry as previously described [18]. Briefly, groups of mice treated with tamoxifen, vehicle or untreated, were infected with *P. berghei* ANKA. Mice were sacrificed 0, 4, and 8 days post-infection, and their spleens were removed and disaggregated using a nylon net. Additionally, three control groups of uninfected mice treated in the same way were used to assess the effects of tamoxifen in the absence of infection. Spleen cells were washed with sterile staining buffer (PBS, 2% SFB, 0.02 NaN_2_) and stained using previously calibrated dilutions of commercial anti-mouse fluorochrome-coupled antibodies: FITC-antiCD3^+^, APC-antiCD4^+^, PE-antiCD8^+^, APC-antiCD19^+^, PE-anti-Mac-3^+^ and PE-antiCD16^+^/32^+^ antibodies. For this purpose, three combinations of antibodies were prepared: The first identified total T cells, T-helper, and cytotoxic T cells (CD3^+^, CD4^+^ and CD8^+^); the second combination identified B cells and macrophages (B220^+^ and anti-Mac-3^+^). The third only contained anti-CD16^+^/32^+^ to identify NK cells. To select the populations, first, the negative and positive region was defined in each dot plot. Three tubes were used for each population: the control of unstained cells, the control of cells stained with a single antibody coupled to the fluorochrome and the tube containing the isotype control. FITC-antiCD3 is a rat IgG2b antibody, and its isotype control was FITC-rat (IGg2b κ); APC-antiCD4 is a rat IgG2a antibody, its isotype control was APC-rat (IgG2a, κ); PE-CD8 is a rat IgG2a antibody, its isotype control was PE-rat (IgG2a, κ); APC-B220 is a rat IgG2a antibody, its isotype control was APC-rat (IgG1, κ); PE-MAC-3 is a rat IgG1 antibody, the isotype control was PE-rat (IgG1, κ). Finally, PE-CD16/32 is an IgG2a antibody, and its isotype control was PE-rat (IgG1 κ). A total of 10,000 cells were evaluated for each sample, and the percentage (%) of positive cells was calculated. All antibodies were purchased from Biolegend (Biolegend, San Diego, CA, USA). Stained cells were analysed using a FACSAria II flow cytometer (Beckton and Dickinson, San Jose, CA, USA); the data were examined with the FlowJo^TM^ software (Beckton and Dickinson, Ashland, OR, USA).

### 4.10. Th1/Th2/Th17 Cytokine Quantification

At 0, 4, and 8 days post-infection, mice were sacrificed, and blood was extracted in heparinised tubes and centrifuged at 2000× *g* for 15 min. The plasma was recovered, aliquoted into two tubes and kept frozen at −20 °C until used. The levels of the cytokines IL-2, IL-4, IL-6, IL-10, TNF-α, IFN-γ and IL-17a were assessed using a cytometric bead array (BD Mouse Th1/Th2/Th17 CBA Kit Biosciences-Pharmingen, Heidelberg, Germany). Briefly, 25 µL of plasma were incubated for 2 h at room temperature in the dark with 25 µL of bead mixture and the detection reagent. The supernatant was separated by centrifugation, and the beads were resuspended in 100 µL of FACS liquid. The concentration of each cytokine was calculated using a commercial standard curve.

### 4.11. Statistical Analysis

Parasitaemia, temperature, body mass variation, and haemoglobin concentration data were analysed by repeated means analysis with a Bonferroni post hoc test and a significance of *p* ≤ 0.05 (*n* = 10). The tamoxifen group was compared against the vehicle-treated and untreated groups on each day. This analysis was performed with the Graph Pad Prism 7 software (Graph Pad Software, San Diego, CA, USA).

The data for splenic index, spleen cell populations and serum cytokine concentration were analysed using the nested treatment effect test 0, 4, and 8 days post-infection with a Bonferroni post hoc test, and significance of *p* ≤ 0.05 (*n* = 10) in each group per day. The nested effect analysis was performed with Statgraphics (Centurion XVI software, The Plains, VA, USA).

## Data Availability

The data presented in this study are available on request from the corresponding author.
